# Ultrafine Diesel Exhaust Particles Induce Apoptosis of Oligodendrocytes by Increasing Intracellular Reactive Oxygen Species through NADPH Oxidase Activation

**DOI:** 10.3390/antiox11051031

**Published:** 2022-05-23

**Authors:** Ji Young Kim, Jin-Hee Kim, Yong-Dae Kim, Je Hoon Seo

**Affiliations:** 1 Department of Anatomy, College of Medicine, Chungbuk National University, Cheongju 28644, Korea; kjyblue84@gmail.com; 2 Department of Biomedical Laboratory Science, College of Health Science, Cheongju University, Cheongju 28503, Korea; jinheekim23273@gmail.com; 3 Department of Preventive Medicine, College of Medicine, Chungbuk National University, Cheongju 28644, Korea; ydkim@cbnu.ac.kr; 4 Chungbuk Regional Cancer Center, Chungbuk National University Hospital, Cheongju 28644, Korea

**Keywords:** air pollution, oxidative stress, gp91phox, NOX2 inhibitor, berberine, demyelinating disease

## Abstract

Diesel exhaust particles (DEPs) are a main contributor to air pollution. Ultrafine DEPs can cause neurodegenerative diseases by increasing intracellular reactive oxygen species (ROS). Compared with other cells in the brain, oligodendrocytes responsible for myelination are more susceptible to oxidative stress. However, the mechanisms underlying ROS generation in oligodendrocytes and the susceptibility of oligodendrocytes to ROS by ultrafine DEPs remain unclear. Herein, we examined the effects of excessive ROS generated by NOX2, an isoform of the NADPH oxidase family, after exposure to ultrafine DEPs (200 μg/mL) on the survival of two types of oligodendrocytes—oligodendrocyte precursor cells (OPCs) and mature oligodendrocytes (mOLs)––isolated from the brain of neonatal rats. In addition, mice were exposed to ultrafine DEP suspension (20 μL, 0.4 mg/mL) via the nasal route for 1 week, after which the expression of NOX2 and cleaved caspase-3 was examined in the white matter of the cerebellum. Exposure to DEPs significantly increased NOX2 expression and ROS generation in OPCs and mOLs. OPCs and mOLs clearly exhibited viability reduction, and a significant change in p53, Bax, Bcl-2, and cleaved caspase-3 expression, after DEP exposure. In contrast, treatment with berberine (BBR), an NOX2 inhibitor, significantly mitigated these effects. In mice exposed to DEP, the presence of NOX2-positive and cleaved caspase-3-positive oligodendrocytes was demonstrated in the cerebellar white matter; NOX2 and cleaved caspase-3 expression in the cerebellum lysates was significantly increased. BBR treatment returned expression of these proteins to control levels. These results demonstrate that the susceptibility of OPCs and mOLs to ultrafine DEPs is, at least in part, caused by excessive ROS produced by NOX2 and the sequential changes in the expression of p53, Bax, Bcl-2, and cleaved caspase-3. Overall, NOX2 inhibitor enhances the survival of two types of oligodendrocytes.

## 1. Introduction

Particulate matter (PM) is a compound of many chemical species and is hazardous to human health. PM is usually categorized according to diameter: PM10 comprises particles with diameters of 2.5–10 μm, whereas PM 2.5 comprises particles with diameters smaller than 2.5 μm [1]. Recently, interest has been increasing in ultrafine PM with diameters smaller than 0.1 μm because smaller particles enter more deeply into the respiratory tract and finally reach the lung [2]. Moreover, smaller particles in the lung can easily enter the bloodstream and reach various organs such as the heart, liver, skin, and brain [1,3,4,5]. In urban areas, a major component of PM is diesel exhaust particles (DEPs); these comprise a significant amount of ultrafine PM associated with traffic emissions [6]. Recent evidence indicates that exposure to DEPs contributes to increases in brain disorders as well as respiratory and cardiovascular diseases [7,8,9,10,11]. DEP-associated brain disorders are mainly caused by oxidative stress. Although reactive oxygen species (ROS) have different effects on cellular functions such as cell differentiation and growth, excessive ROS generation may lead to oxidative stress-induced apoptosis of brain cells and contribute to the onset of brain disorders such as Parkinson’s disease, cognitive impairment, Alzheimer’s disease, and multiple sclerosis [12].

Nicotinamide adenine dinucleotide phosphate (NADPH) oxidase is a transmembrane protein complex with multisubunits; it catalyzes the molecular oxygen reduction and the oxidation of NADPH to produce superoxide radicals [13]. Superoxide radicals generated by NADPH oxidase (NOX) contribute to defense against infection in phagocytic cells and enhance physiological processes, including the regulation of hormones and factors in nonphagocytic cells [14]. NOX2 (gp91phox), an isoform of the NADPH oxidase family, has recently become known as a major contributor to ROS generation by DEPs in brain cells [15]. Furthermore, oxidative stress caused by excessive ROS generation increases the expression of p53, which mediates apoptosis through Bax and caspase upregulation and Bcl-2 suppression [16]. Consequently, oxidative stress induces p53-dependent apoptosis of brain cells and acts as a major contributor to brain disorders [17].

Oligodendrocytes are myelin-forming cells present in the central nervous system (CNS). These cells are known to be susceptible to oxidative stress [18]. They have a poor ability to scavenge ROS due to their low antioxidant capacity [19]. Our previous studies demonstrated that unlike any other brain cells, oligodendrocytes were significantly damaged by ROS induced by hydrogen peroxide [20,21]. The facts that ROS levels were significantly elevated in rodents with clinical signs of experimental allergic encephalomyelitis (EAE) and that the treatment of ROS scavengers suppressed the severity of EAE reflects that the viability of oligodendrocytes is closely related to oxidative stress [22]. However, the mechanisms underlying ROS generation in oligodendrocytes and the susceptibility of oligodendrocytes to ROS by ultrafine DEPs remain unclear. Herein, we examined whether excessive ROS produced by NOX2 after exposure to ultrafine DEPs damage oligodendrocyte precursor cells (OPCs) and mature oligodendrocytes (mOLs) and whether NOX2 inhibitor enhances the survival of OPCs and mOLs.

## 2. Materials and Methods

### 2.1. Culturing of Rodent Brain Cells

We isolated and cultured each cell type according to the methods elucidated in our previous study [23]. In brief, we obtained the mixed neuroglial cells from neonate Sprague-Dawley rats (DBL, Eumseong, Korea). We incubated the mixed neuroglial cells in the flask in a shaking incubator for 1 h at 200 rpm. After disposal of the media containing microglia, we incubated the flask in a shaking incubator at 200 rpm for 18–20 h to isolate OPCs. We plated the media containing OPCs and maintained the OPCs in OPC medium for 10 days in a 5% CO_2_ incubator. We detached astrocytes on the bottom of the flask with trypsin-ethylenediaminetetraacetic acid and maintained the astrocytes in astrocyte medium for 10 days in a 5% CO_2_ incubator. To obtain mOLs, we differentiated OPCs to mOLs by maintaining the OPCs in mOL medium for 7–9 days in a 5% CO_2_ incubator. To obtain cortical neurons, we maintained the dissociated cells from the cerebra of neonatal rats in neurobasal-A medium for 7 days in a 5% CO_2_ incubator.

### 2.2. Identification of Brain Cell Types

To identify two types of oligodendrocytes, astrocytes, and cortical neurons, we examined the shape and phenotypical antigenicity of each cell. Nine days after initiating the culture, we observed the morphology of cells under a phase-contrast microscope (CK40, Olympus, Tokyo, Japan). We performed immunofluorescence staining using cell-specific antibodies: anti-A2B5 antibody for OPCs, anti-myelin basic protein (MBP) antibody for mOLs, anti-glial fibrillary acidic protein (GFAP) antibody for astrocytes, and anti-NeuN antibody for cortical neurons. To assess the purity of isolated cells, we calculated the proportions of cells stained with specific markers among total cells counterstained with 4′,6-diamidino-2-phenylindole (DAPI).

### 2.3. Preparation and Exposure to a Working Suspension of Ultrafine DEPs and NOX2 Inhibition

We prepared working suspensions of ultrafine DEPs according to the previous method [24]. In brief, we suspended 2 mg of DEPs (NIST2975, Sigma, St. Louis, MO, USA) in 10 mL phosphate-buffered saline (PBS). We vortexed, sonicated, and filtered DEPs in PBS through a syringe filter (0.2-µm; Sartorius, Goettingen, Germany). For various concentrations of working suspensions (2, 20, 200 µg/mL), we resuspended ultrafine DEPs in a serum-free culture medium. We subjected each cell type to the working suspensions for 24 h in a 5% CO_2_ incubator. For the control, we also subjected each cell type to a serum-free culture medium without ultrafine DEPs in the same condition. To inhibit NOX2 activity, we used berberine (BBR), an inhibitor of NOX2. We pretreated BBR (10 μM; Sigma, St. Louis, MO, USA) to each cell type for 1 h before exposure to ultrafine DEPs. We performed all measurements immediately after exposure of ultrafine DEPs.

### 2.4. Measurement of ROS Generation

We performed the dichlorofluorescein (DCF) assay and dihydroethidine (DHE) staining to measure ROS generation [25,26]. For the DCF assay, we incubated each cell type with 2ʹ,7ʹ-dichlorofluorescein diacetate (DCFH-DA; 100 μM) in the medium for 30 min in a 5% CO_2_ incubator and measured the cell fluorescence from each well using SpectraMax M5*^e^* (Molecular Devices, San Jose, CA, USA). For DHE staining, we incubated each cell type with 10 μM DHE in a cell medium for 30 min in a 5% CO_2_ incubator. We observed stained cells under a multipurpose microscope equipped with an epifluorescence attachment (DMLB, Leica, Wetzlar, Germany).

### 2.5. Measurement of Cell Viability

We performed the 3-(4,5-dimethylthiazol-2-yl)-2,5-diphenyltetrazolium bromide (MTT) assay for the measurement of cell viability. We added MTT salts (10 μL of 5 mg/mL; Sigma, St. Louis, MO, USA) to each cell type for 4 h. Then, we added 110 μL of solubilization buffer (27 mL isopropanol, 3 mL Triton X-100, 2.5 μL HCl) to each well and incubated the plate in a shaking incubator at room temperature for 10 min. To determine optical density values, we measured absorbance at 570 nm using a spectrophotometer (Bio-Rad, Hercules, CA, USA).

### 2.6. Measurement of Apoptosis

We performed the annexin V assay for the measurement of apoptosis using Muse Annexin V and Dead Cell Kit (Millipore, Billerica, MA, USA), according to the manufacturer’s instructions. We analyzed the relative percentage of total apoptotic cells using a Muse Cell Analyzer (Millipore, Billerica, MA, USA).

### 2.7. Hoechst Staining to Identify Dead Cells

We fixed each cell type with 4% paraformaldehyde (PFA) for 20 min and washed in PBS twice for 5 min each. We incubated the cells with Hoechst 33,258 solution (2 μg/mL; Sigma, St. Louis, MO, USA) for 15 min at 37 °C and rinsed with PBS. We observed the stained cells under a multipurpose microscope equipped with an epifluorescence attachment (DMLB, Leica, Wetzlar, Germany). We determined cells with condensed or fragmented nuclei in the Hoechst staining images as dead cells.

### 2.8. Animals and Exposure to Ultrafine DEPs

We commercially purchased 7-week-old male Balb/c mice (20–23 g, *n* = 9; DBL, Eumseong, Korea) and housed the mice under standard laboratory conditions under a 12 h light/dark cycle at 24–26 °C with a commercial diet and water ad libitum. We allocated the mice into three groups: control (*n* = 3), ultrafine DEP exposure group (DEP; *n* = 3), and ultrafine DEP exposure plus BBR treatment group (DEP + BBR; *n* = 3). Before exposure to ultrafine DEPs, we lightly anesthetized the mice with isoflurane and gently instilled 20 μL of the ultrafine DEP solution (0.4 mg/mL in PBS) into the nasal cavities of the mice using a micropipette in the supine position. We exposed ultrafine DEPs to the mice in the DEP and DEP + BBR groups twice a day in 12 h intervals for 5 days consecutively and once a day in 24 h intervals for 2 days consecutively. We intraperitoneally injected 100 µL of BBR (0.05 mg/kg; Sigma, St. Louis, MO, USA) into the mice in the DEP + BBR group once a day for 7 days consecutively. We instilled 20 μL of PBS in the mice in the control group instead of the ultrafine DEP solution.

### 2.9. Preparation of Tissue

On the day after final instillation, we anesthetized the mice with ketamine hydrochloride (100 mg/kg; Yuhan Co., Seoul, Korea) and xylazine hydrochloride (10 mg/kg; Bayer Korea, Seoul, Korea). We transcardially perfused the anesthetized mice with precooled saline and 4% PFA and excised the brains. After postfixation for 8 h, we dehydrated the brain in 30% sucrose in PBS overnight for cryoprotection. We embedded the brain tissues in optimal cutting temperature compound (Leica, Wetzlar, Germany). We rapidly froze the brain tissues in 2-methyl butane (Junsei, Tokyo, Japan) adjusted to its freezing point with liquid nitrogen and sectioned the tissues to a thickness of 12 μm using a cryostat (CM3050S, Leica, Wetzlar, Germany). We then placed the sectioned tissues on gelatin-coated slide glasses.

### 2.10. Immunofluorescence Staining

We incubated the cells and brain sections on slides in PBS with 10% normal goat serum for 30 min. We then incubated the cells and sections overnight at 4 °C with mouse anti-A2B5 (1:5, #mAB4D4, Developmental Studies Hybridoma Bank, Iowa City, IA, USA), rat anti-MBP (1:500, #MAB386, Millipore, Burlington, MA, USA), rabbit anti-GFAP (1:200, #Z0334, Dako, Santa Clara, CA, USA), mouse anti-NeuN (1:100, #MAB377, Chemicon, Gaithersburg, MD, USA), mouse anti-gp91phox (1:100, #sc-130543, Santa Cruz, Dallas, TX, USA), rabbit anti-cleaved caspase-3 (1:200, #9664, Cell Signaling, Danvers, MA, USA), rabbit anti-carbonic anhydrase II (CAII, 1:200, #ab124687, Abcam, Cambridge, UK), and mouse anti-αB crystallin (aBC, 1:10, #CPTC-CRYAB-3, Developmental Studies Hybridoma Bank, Iowa City, IA, USA) antibodies. Next, we incubated the cells and sections for 2 h at room temperature with Cy2-labeled goat anti-rat IgG (1:500, Jackson ImmunoResearch Laboratories, West Grove, PA, USA), Cy2-labeled goat anti-rabbit IgG (1:200, Jackson ImmunoResearch Laboratories, West Grove, PA, USA), and Cy2-labeled horse anti-mouse IgG (1:200, Jackson ImmunoResearch Laboratories, West Grove, PA, USA). Between each step, we washed the cells and sections 3 times with PBS for 10 min. Finally, we observed the stained cells and sections under a multipurpose microscope equipped with an epifluorescence attachment (DMLB, Leica, Wetzlar, Germany).

### 2.11. Western Blot

We lysed each cell type at 4 °C for 20 min in radioimmunoprecipitation assay buffer (Elpis-Biotech, Daejeon, Korea). We centrifuged lysates at 14,000 rpm for 5 min at 4 °C and denatured the samples by boiling in sample buffer for 5 min. Then, we separated the samples on a 10%–15% Tris–HCl gel using electrophoresis. We transferred the separated proteins onto polyvinylidene difluoride membranes (Millipore, Burlington, MA, USA). After blocking with 5% skim milk in PBS for 1 h, we incubated the membranes with primary antibodies against gp91phox (1:100, #sc-130543, Santa Cruz, Dallas, TX, USA), p53 (1:100, #sc-99, Santa Cruz, Dallas, TX, USA), Bax (1:500, #14796, Cell Signaling, Danvers, MA, USA), Bcl-2 (1:500, #ab7973, Abcam, Cambridge, UK), cleaved caspase-3 (1:500, #9664, Cell Signaling, Danvers, MA, USA), and actin (1:10, #JLA20, Developmental Studies Hybridoma Bank, Iowa City, IA, USA). We incubated the membranes with secondary antibodies, including peroxidase-conjugated horse anti-mouse IgG (1:500, Vector Laboratories, Burlingame, CA, USA) and peroxidase-conjugated goat anti-rabbit IgG (1:1000, Vector Laboratories, Burlingame, CA, USA). Between each step, we intensely washed the membranes 3 times with PBS for 10 min. We detected the immunoreactive bands using 4IPBA-ECL solution [27] and a ChemiDoc (Bio-Rad, Hercules, CA, USA).

### 2.12. Statistical Analysis

We expressed data as mean ± standard error of the mean. We calculated statistical differences using one-way ANOVA followed by Bonferroni analysis of variance with the Prism 5 program (GraphPad, San Diego, CA, USA). We considered *p* < 0.05 is statistically significant.

## 3. Results

### 3.1. Identification of Brain Cell Types

**Figure 1:**
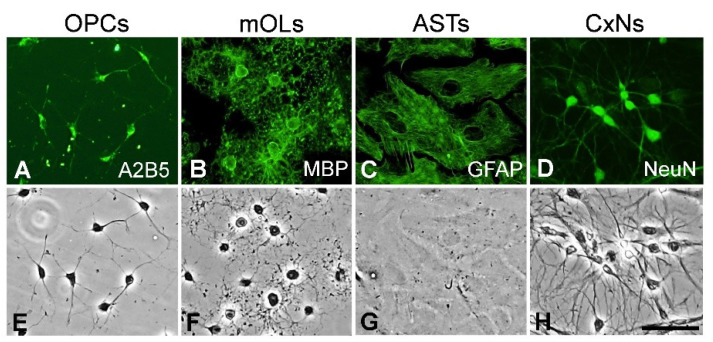
The expression of specific antigens and morphology of primary cells isolated from the brains of neonatal rats. (**A**–**D**) Specific antigen expression. OPCs, mOLs, astrocytes, and cortical neurons are labeled with specific antibodies. (**E**–**H**) Morphology. The unique shapes of OPCs, mOLs, astrocytes, and cortical neurons are visualized using phase-contrast microscopy. ASTs = astrocytes, CxNs = cortical neurons. Scale bar = 50 μm.

We identified the specific antigen expression and typical morphology of two types of oligodendrocytes (OPCs and mOLs), astrocytes, and cortical neurons using immunofluorescence and phase-contrast microscopy. A2B5-positive OPCs were ovoid in shape and had bipolar processes (Figure 1A,E), whereas MBP-positive mOLs had spherical cell bodies with fine multipolar processes (Figure 1B,F). GFAP-positive astrocytes had large cell bodies that were polygonal (Figure 1C,G). NeuN-positive cortical neurons had fusiform or pyramidal cell bodies with long, thin processes (Figure 1D,H). Among total cells stained with DAPI, the proportions of A2B5-positive OPCs, MBP-positive mOLs, GFAP-positive astrocytes, and NeuN-positive cortical neurons were 97%, 92%, 98%, and 96%, respectively (Supplementary Figure S1). These results reveal that the procedures for culturing of each primary cell type were performed accurately.

### 3.2. ROS Generation According to Concentration of Ultrafine DEPs

**Figure 2:**
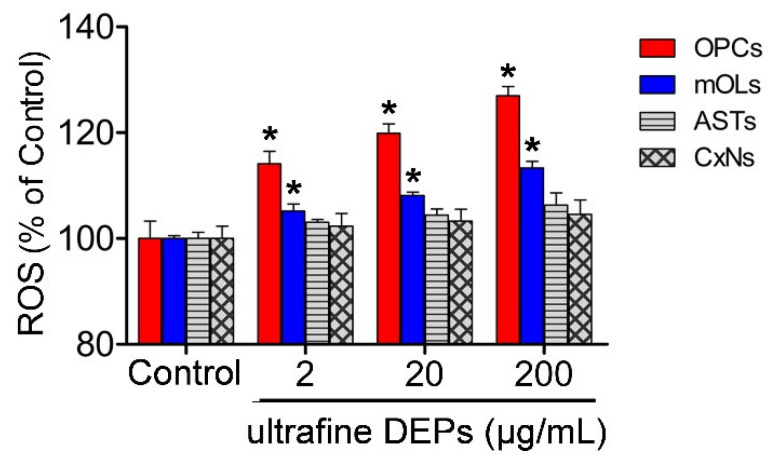
Detection of ROS generation using the DCF assay in brain cells after exposure to various concentrations of ultrafine DEPs (2, 20, 200 μg/mL). Compared with the control, the ROS levels are significantly increased in OPCs and mOLs at 2 μg/mL and tend to be increased in proportion to the ultrafine DEP concentrations. Note that the ROS levels of astrocytes and cortical neurons are not changed after exposure to ultrafine DEP. Cont = control, ASTs = astrocytes, CxNs = cortical neurons. * *p* < 0.05 vs. control.

To examine the changes in ROS generation in brain cells according to the various concentrations of ultrafine DEPs, we measured the ROS levels using the DCF assay at 2, 20, and 200 μg/mL concentrations of ultrafine DEP. Compared with the control, the ROS levels of the OPCs and mOLs were significantly increased at every concentration of ultrafine DEPs we measured (Figure 2). The ROS levels of OPCs and mOLs tended to be increased in proportion compared with the ultrafine DEP concentration. At 200 μg/mL of ultrafine DEPs, the ROS level of OPCs was increased to up to 27% compared with the control. However, there was no change in the ROS levels of astrocytes and cortical neurons at any concentration of ultrafine DEPs.

### 3.3. Gp91phox Expression after Exposure to Ultrafine DEPs

**Figure 3:**
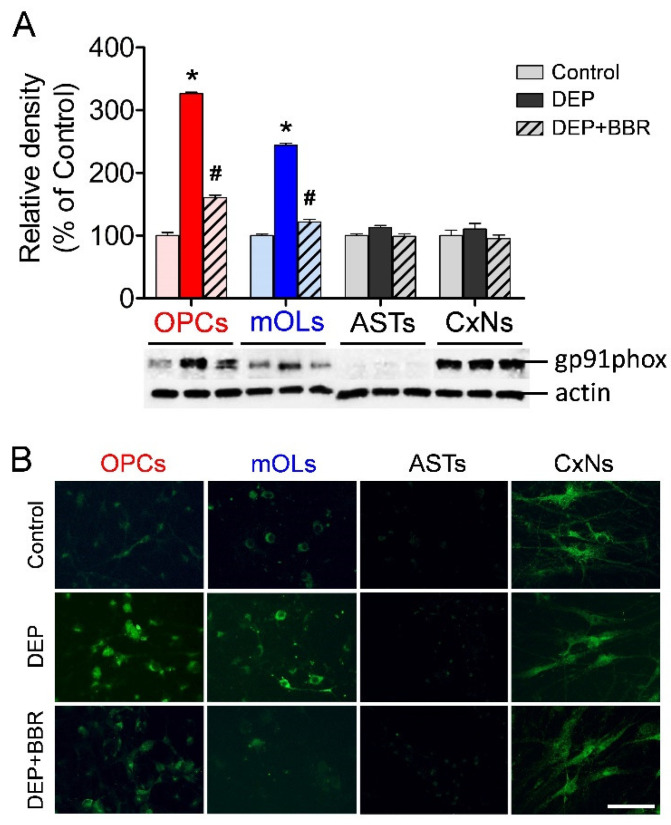
Expression of gp91phox (NOX2) in brain cells after exposure to ultrafine DEPs. (**A**) Quantitative analysis. The gp91phox expressions in OPCs and mOLs exposed to ultrafine DEPs (200 μg/mL) are significantly higher than those of each control group. However, BBR treatment significantly suppresses the gp91phox expressions in OPCs and mOLs exposed to ultrafine DEPs. There are no differences in the gp91phox expressions of astrocytes and cortical neurons in each group. (**B**) Immunofluorescence. The gp91phox expressions in OPCs and mOLs exposed to ultrafine DEPs are markedly increased compared with those of each control group. However, BBR treatment suppresses the gp91phox expressions in OPCs and mOLs exposed to ultrafine DEPs. Changes in gp91phox expressions are not observed in astrocytes and cortical neurons in each group. ASTs = astrocytes, CxNs = cortical neurons. * *p* < 0.05 for DEP group vs. control, # *p* < 0.05 for DEP + BBR group vs. DEP group. Scale bar = 200 μm.

To examine the changes in NOX2 expression in brain cells after exposure to ultrafine DEPs (200 μg/mL), we performed a Western blot and immunofluorescence staining using a primary antibody against gp91phox. The expressions of gp91phox were significantly increased in OPCs and mOLs after exposure to ultrafine DEPs (Figure 3A). In contrast, BBR treatment significantly suppressed gp91phox expression in OPCs and mOLs after exposure to ultrafine DEPs. The results of immunofluorescence staining were completely consistent with those of the Western blot. The gp91phox expressions were remarkably increased in OPCs and mOLs after exposure to ultrafine DEPs and suppressed upon BBR treatment in OPCs and mOLs exposed to ultrafine DEPs (Figure 3B). Although gp91phox expression was absent in astrocytes and abundant in cortical neurons in the control, there were no significant differences in the gp91phox expressions of astrocytes and cortical neurons after exposure to ultrafine DEPs and BBR treatment (Figure 3A,B).

### 3.4. ROS Generation by NOX2 after Exposure to Ultrafine DEPs

**Figure 4:**
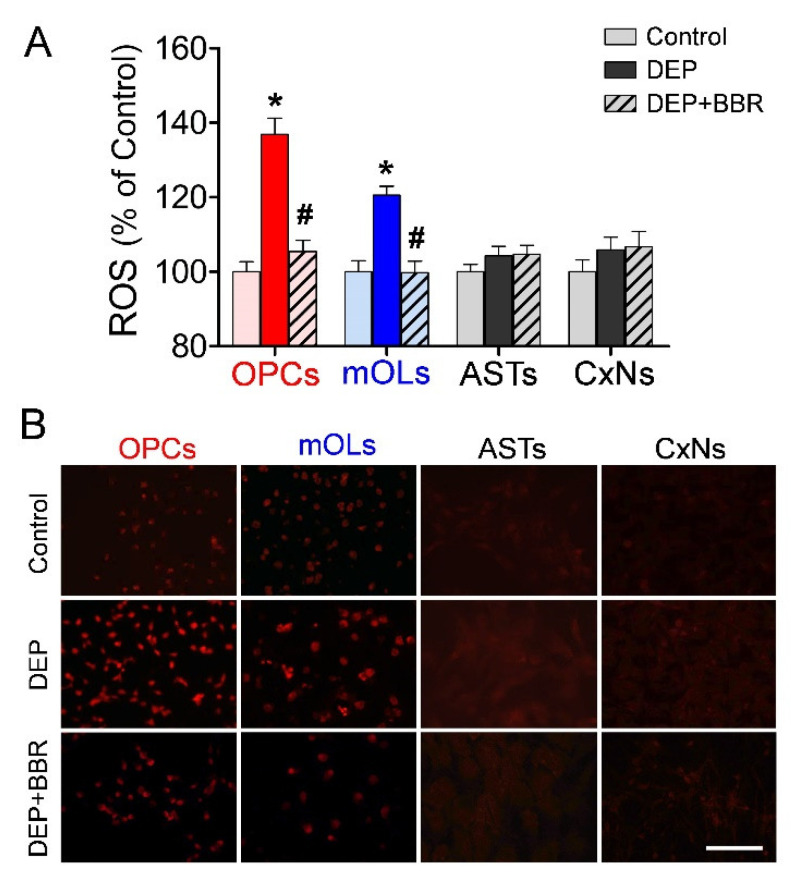
Detection of ROS generation in brain cells after exposure to ultrafine DEPs. (**A**) DCF assay. ROS generation in OPCs and mOLs after exposure to ultrafine DEP (200 μg/mL) is significantly increased compared with that in each control group. However, BBR treatment significantly inhibits ROS generation in OPCs and mOLs exposed to ultrafine DEPs. (**B**) DHE staining. ROS generation after exposure to ultrafine DEPs is markedly increased in OPCs and mOLs, whereas ROS generation is not observed in astrocytes and cortical neurons in each group. ASTs = astrocytes, CxNs = cortical neurons. * *p* < 0.05 for DEP group vs. control, # *p* < 0.05 for DEP + BBR group vs. DEP group. Scale bar = 200 μm.

To examine the changes in ROS generation due to NOX2 in brain cells after exposure to ultrafine DEPs (200 μg/mL), we performed a DCF assay and DHE staining. The DCF assay demonstrated that ROS generation was significantly elevated after exposure to ultrafine DEPs (Figure 4A). In contrast, BBR treatment significantly inhibited ROS generation in OPCs and mOLs exposed to ultrafine DEPs to the level of the control. The results of the DHE staining were consistent with those of the DCF assay. ROS generation was remarkably increased in OPCs and mOLs after exposure to ultrafine DEPs and inhibited by BBR treatment in OPCs and mOLs exposed to ultrafine DEPs (Figure 4B). However, the level of ROS generation in astrocytes and cortical neurons was quite low in the control, and an increase in ROS generation was not observed even after exposure to ultrafine DEPs (Figure 4A,B).

### 3.5. Expression of p53, Bax, Bcl-2, and Cleaved Caspase-3 after Exposure to Ultrafine DEPs

**Figure 5:**
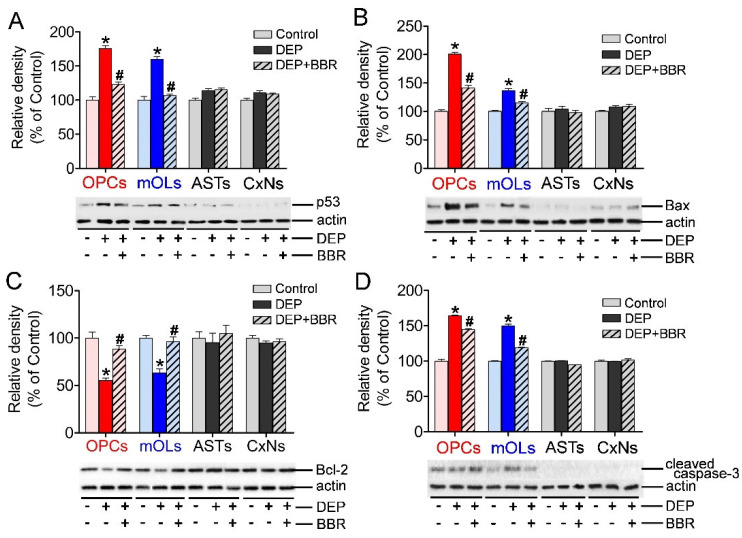
Expression of p53, Bax, Bcl-2, and cleaved caspase-3 in brain cells after exposure to ultrafine DEP. The expressions of p53 (**A**), Bax (**B**), and cleaved caspase-3 (**D**) are significantly increased and the expression of Bcl-2 (**C**) is significantly decreased in OPCs and mOLs after exposure to ultrafine DEPs (200 μg/mL) compared with those in the control groups. However, BBR treatment significantly suppresses the expressions of p53 (**A**), Bax (**B**), and cleaved caspase-3 (**D**) and recovers the expression of Bcl-2 **(C)** in OPCs and mOLs exposed to ultrafine DEPs. There are no differences in the expressions of p53 (**A**), Bax (**B**), Bcl-2 (**C**), and cleaved caspase-3 (**D**) in astrocytes and cortical neurons in each group. ASTs = astrocytes, CxNs = cortical neurons. * *p* < 0.05 for DEP group vs. control, # *p* < 0.05 for DEP + BBR group vs. DEP group.

To determine whether ROS produced by NOX2 activated p53-dependent apoptosis, we examined the expressions of p53, Bax, Bcl-2, and cleaved caspase-3 in brain cells after exposure to ultrafine DEPs (200 μg/mL). The expressions of p53, Bax, and cleaved caspase-3 were significantly increased and the expression of Bcl-2 was significantly decreased in OPCs and mOLs after exposure to ultrafine DEPs (Figure 5A–D). In contrast, BBR treatment significantly suppressed the expressions of p53, Bax, and cleaved caspase-3 and recovered the expression of Bcl-2 in OPCs and mOLs exposed to ultrafine DEPs (Figure 5A–D). However, there were no changes in the expressions of p53, Bax, Bcl-2, and cleaved caspase-3 in astrocytes and cortical neurons after exposure to ultrafine DEPs and BBR treatment. These results suggest that ROS produced by NOX2 activate p53-dependent apoptosis of OPCs and mOLs but not that of astrocytes and cortical neurons.

### 3.6. Decrease in Cell Viability after Exposure to Ultrafine DEPs

**Figure 6:**
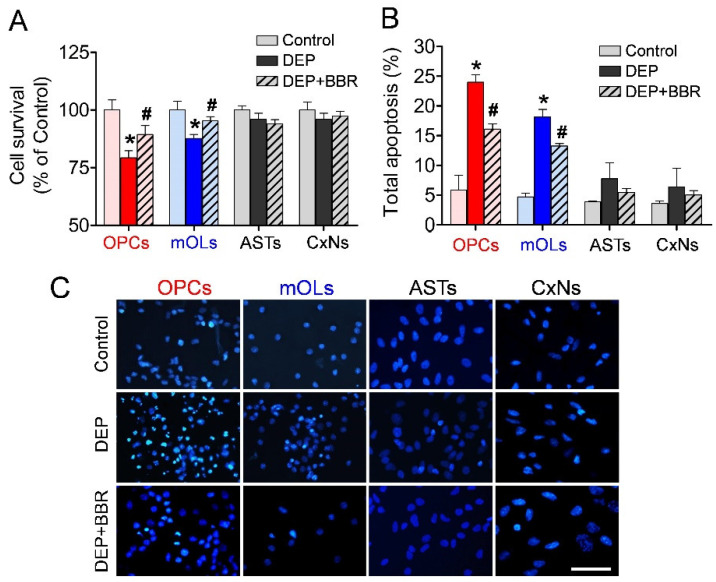
Viability analyses of brain cells after exposure to ultrafine DEP. (**A**) MTT assay. The survival rates of OPCs and mOLs exposed to ultrafine DEPs (200 μg/mL) are significantly decreased compared with those in each control group. BBR treatment inhibits the significant decrease of the survival rates of OPCs and mOLs exposed to ultrafine DEPs. There are no significant changes in the survival rates of astrocytes and cortical neurons compared with that in each control group. (**B**) Annexin V assay. The total proportions of apoptotic OPCs and mOLs are significantly increased compared with those in each control group. BBR treatment inhibits the significant increase of the total proportions of apoptotic OPCs and mOLs exposed to ultrafine DEPs. Although the total proportions of apoptotic astrocytes and cortical neurons are slightly elevated, there are no statistical changes compared with those in each control group. (**C**) Hoechst staining. The number of damaged OPCs and mOLs with condensed or fragmented nuclei after ultrafine DEP exposure (200 μg/mL) are markedly increased compared with that in each control group. However, BBR treatment inhibits the increase of the number of damaged OPCs and mOLs exposed to ultrafine DEPs. Damaged astrocytes and cortical neurons are not observed in the three groups. ASTs = astrocytes, CxNs = cortical neurons. * *p* < 0.05 for DEP group vs. control, # *p* < 0.05 for DEP + BBR group vs. DEP group. Scale bar = 200 μm.

To examine the viability and damage of brain cells after exposure to ultrafine DEPs (200 μg/mL), we performed MTT and annexin V assays and Hoechst staining. The MTT assay demonstrated that ultrafine DEPs significantly decreased the viability of OPCs and mOLs compared with the control (Figure 6A). In contrast, BBR treatment inhibited the significant reduction of the viability of OPCs and mOLs exposed to ultrafine DEPs. However, there were no changes in the viability of astrocytes and cortical neurons after exposure to ultrafine DEPs and BBR treatment. The annexin V assay also demonstrated that the total proportions of damaged OPCs and mOLs were significantly increased compared with the controls (Figure 6B). Furthermore, BBR treatment inhibited the significant increase of the total proportions of damaged OPCs and mOLs exposed to ultrafine DEPs. Although the total proportions of damaged astrocytes and cortical neurons were slightly increased after exposure to ultrafine DEPs, there were no statistically significant differences between the DEP and control groups. Hoechst staining also demonstrated that the number of OPCs and mOLs with fragmented and condensed nuclei were remarkably increased after exposure to ultrafine DEPs (Figure 6C). Furthermore, BBR treatment inhibited the increase of the number of damaged OPCs and mOLs exposed to ultrafine DEPs. However, there were no changes in the numbers of damaged astrocytes and cortical neurons after exposure to ultrafine DEPs and BBR treatment. These results suggest that the inhibition of NOX2 activity selectively suppresses the ultrafine DEP-induced oxidative damage of OPCs and mOLs.

### 3.7. Damage of gp91phox-Positive Oligodendrocytes in Cerebellar White Matter

**Figure 7:**
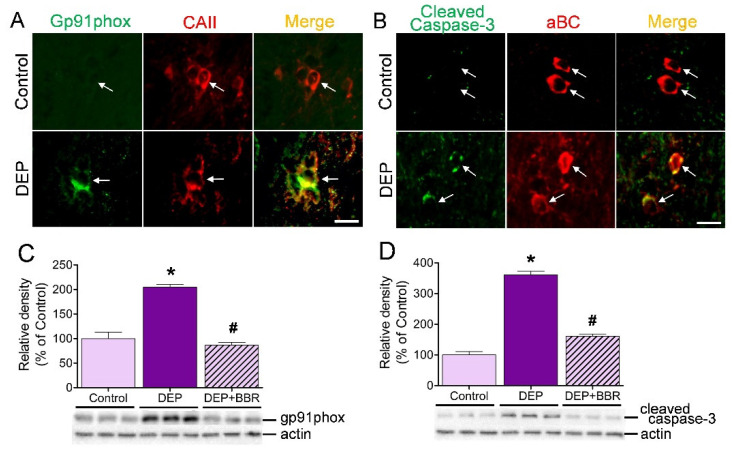
Expressions of gp91phox (NOX2) and cleaved caspase-3 in the cerebellum of mice exposed to ultrafine DEPs for 1 week. (**A**,**B**) Double immunofluorescence staining. Unlike the control, the CAII-positive and aBC-positive oligodendrocytes overlapped with the gp91phox (**A**) and cleaved caspase-3 (**B**) are found in the cerebellar white matter of the mouse brain exposed to ultrafine DEPs. (**C**,**D**) Quantitative analysis. The expressions of gp91phox (**C**) and cleaved caspase-3 (**D**) in the mouse cerebellum exposed to ultrafine DEPs are significantly higher than those in each control group. However, BBR treatment significantly suppressed the expressions of gp91phox and cleaved caspase-3 in the cerebellum exposed to ultrafine DEPs. * *p* < 0.05 for DEP group vs. control, # *p* < 0.05 for DEP + BBR group vs. DEP group. Scale bar = 10 μm.

To determine whether ultrafine DEPs damage gp91phox-positive oligodendrocytes in the cerebellar white matter of mice, we performed double immunofluorescence staining and a Western blot. Double immunofluorescence staining clearly demonstrated that unlike the case of the control group, numerous oligodendrocytes were overlapped with gp91phox (Figure 7A) and cleaved caspase-3 (Figure 7B) in the cerebellar white matter after one-week exposure of mice to ultrafine DEPs. The Western blot demonstrated that exposure to ultrafine DEPs significantly increased the expressions of gp91phox and cleaved caspase-3 in the cerebellum of the DEP group than in that of the control group (Figure 7C,D). BBR treatment, however, significantly suppressed the expressions of gp91phox and cleaved caspase-3 compared with that in each DEP group. These results suggest that exposure to ultrafine DEP causes damage to cerebellar oligodendrocytes and that the damage is suppressed by NOX2 inhibitors in mice.

## 4. Discussion

Several previous studies have demonstrated that ultrafine PM reaches the brain presumably via the olfactory nerves and blood circulation [28]. NOX activation is considered one of the main causes for ROS generation by ultrafine PM in the brain. Investigations on the neurotoxicity of NOX2 have focused on microglia, which are phagocytotic cells in the CNS. NOX2 overexpression in activated microglia induces the release of superoxide radicals and stimulates the secretion of proinflammatory cytokines via cell signaling by ROS, thereby leading to damage of neighboring neurons [17]. Similarly, several studies on NOX2 function, right from physiological metabolism to cell death, have been conducted on neurons and astrocytes [17]. However, little information is available on the expression and role of NOX2 on oligodendrocytes, the sole myelinating cells in the CNS, despite the fact that the possibility of deep correlation between multiple sclerosis and oxidative stress induced by ultrafine PM has been steadily increasing [29]. In the present study we demonstrated that ultrafine DEPs significantly increased NOX2 expression in OPCs and mOLs. Furthermore, increased NOX2 produced excessive ROS in OPCs and mOLs. Consequently, the excessive ROS damaged the OPCs and mOLs, similar to the results of previous studies in cardiomyocytes, lung epithelial cells, and keratinocytes [4,5,30]. Interestingly, although NOX2 expression of cortical neurons was high with or without ultrafine DEPs, the ROS level remained low. The low level of ROS resulted in no significant damage to cortical neurons. These findings suggest that cortical neurons have a well-functioning antioxidant system for scavenging ROS. On the other hand, oligodendrocytes have a low antioxidant capacity [19,31]. Our previous study also demonstrated that OPCs and mOLs are more susceptible to oxidative stress induced by ultrafine urban particles owing to them having a lower total antioxidant capacity than astrocytes and neurons [23]. Therefore, the low levels of intracellular antioxidants also seem to be a critical cause of severe toxicity to oxidative stress in OPCs and mOLs.

Oxidative stress by excessive intracellular ROS induces increases in p53, a transcription factor related to apoptosis [32]. p53 enhances the expression of proapoptotic Bcl-2 family members such as Bax, which allows the release of cytochrome c from the mitochondria [33]. p53 also suppresses the expression of Bcl-2, an antiapoptotic Bcl-2 member that inhibits Bax from releasing cytochrome c [16]. Finally, caspases are cleaved and apoptosis progresses [34]. Air pollutants reportedly increase p53-dependent apoptosis in various cells. DEP exposure increases p53 expression, resulting in the apoptosis of alveolar epithelial cells and keratinocytes [35,36]. Fine PM also induces the apoptosis of endothelial cells and macrophages by increasing p53 activation, which increases the expression of Bax and caspase-9, -7, and -3 [37]. Similar to previous investigations, we demonstrated in the present study that the expressions of p53, Bax, and caspase-3 were significantly increased and that the expression of Bcl-2 was significantly decreased in OPCs and mOLs, but not in astrocytes and cortical neurons, after exposure to ultrafine DEP. Our findings highlight the possibility that the inhibition of p53-dependent apoptosis could be useful in the selective survival of oligodendrocytes.

Various inhibitors that suppress NOX function have been developed for clinical implications. NOX inhibition reduced the clinical features and neuropathological changes associated with EAE-induced white matter damage in mice [38]. Furthermore, treatment with an inhibitor after NOX activation upon exposure to lipopolysaccharide prevented microglial toxicity to oligodendrocytes [39]. BBR is a naturally occurring isoquinoline alkaloid with anticardiovascular and anticancer properties [40]. Similar to the findings of a previous report that BBR selectively inhibits the mRNA expression of the main subunit of NOX2 in lipopolysaccharide-stimulated macrophages [41], our results show that BBR significantly inhibits NOX2 function inducing ROS generation after exposure to ultrafine DEPs, resulting in significant reduction of OPC and mOL cell death. In addition to this direct effect of NOX2 on oligodendrocytes, BBR might attenuate the toxicity against oligodendrocytes by reducing superoxide generation from macrophages and microglia [41]. Taken together, BBR is potentially useful in the development of therapeutics for oligodendrocyte-related diseases such as multiple sclerosis in the future.

## 5. Conclusions

The susceptibility of OPCs and mOLs to ultrafine DEPs is, at least in part, caused by excessive ROS produced by NOX2, and sequential increases in the expressions of p53, Bax, and cleaved caspase-3 and decreases in Bcl-2 expression. Furthermore, NOX2 inhibitor enhances the survival of OPCs and mOLs. These findings provide an improved understanding of the mechanisms underlying the damage of oligodendrocytes caused by ultrafine DEPs, which could potentially be useful in future clinical applications.

## Data Availability

All data are contained within this article.

## Supplementary Materials

The following are available online at https://www.mdpi.com/article/10.3390/antiox11051031/s1. Figure S1. High proportions of isolated cells stained with cell-specific markers.
