# Supplementary material for: Ultrafine Diesel Exhaust Particles Induce Apoptosis of Oligodendrocytes by Increasing Intracellular Reactive Oxygen Species through NADPH Oxidase Activation
Source: Antioxidants (Basel). 2022 May 23;11(5):1031. doi: 10.3390/antiox11051031 (PMC9137819; doi:10.3390/antiox11051031)
Supplement: Supplementary file 1 [file antioxidants-11-01031-s001.zip › antioxidants-1711182-supplementary.pdf]

Supplementary Figure

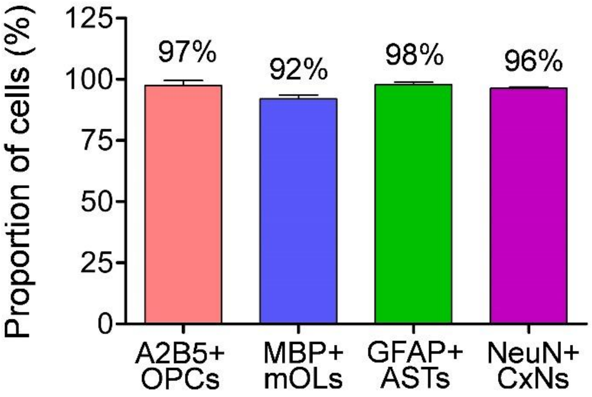

Figure S1. High proportions of isolated cells stained with cell-specific markers. The proportions of A2B5-positive OPCs, MBP-positive mOLs, GFAP-positive astrocytes, and NeuN-positive cortical neurons are 97%, 92%, 98%, and 96% among total cells counterstained with DAPI-positive cells, respectively.
